# Relationship Between Cerebral Hemodynamics, Tissue Oxygen Saturation, and Delirium in Patients With Septic Shock: A Pilot Observational Cohort Study

**DOI:** 10.3389/fmed.2021.641104

**Published:** 2021-11-26

**Authors:** Qing Feng, Meilin Ai, Li Huang, Qianyi Peng, Yuhang Ai, Lina Zhang

**Affiliations:** ^1^Department of Intensive Care Unit, Central South University, Xiangya Hospital, Changsha, China; ^2^Department of Intensive Care Unit, Peking University, Shenzhen Hospital, Shenzhen, China

**Keywords:** sepsis, septic shock, transcranial Doppler ultrasound, cerebral oxygen saturation, tissue oxygen saturation, delirium, sepsis-associated delirium

## Abstract

**Background:** Septic shock patients have tendencies toward impairment in cerebral autoregulation and imbalanced cerebral oxygen metabolism. Tissue Oxygen Saturation (StO_2_) and Transcranial Doppler (TCD) monitoring were undertaken to observe the variations of cerebral hemodynamic indices and cerebral/peripheral StO_2_ to find risk factors that increase the sepsis-associated delirium (SAD).

**Materials and Methods:** The research cohort was chosen from septic shock patients received in the Department of Critical Care Medicine, Xiangya Hospital, Central South University between May 2018 and March 2019. These patients were separated into two groups, SAD and non-SAD as assessed by using the Confusion Assessment Method for the Intensive Care Unit (CAM-ICU). Comparisons were made between the two groups in terms of peripheral StO_2_, fluctuations in regional cerebral oxygen saturation (rSO_2_), cerebral vascular automatic regulation function [Transient Hyperemic Response Ratio (THRR) index], cerebral hemodynamic index, organ function indicators, blood gas analysis indices, and patient characteristics.

**Results:** About 39% of the patients (20/51) suffered from SAD. Nearly 43% of the patients died within 28 days of admission (22/51). Individuals in the SAD cohort needed a longer period of mechanical ventilation [5 (95% CI 2, 6) vs. 1 days (95% CI 1, 4), *p* = 0.015] and more time in ICU [9 (95% CI 5, 20) vs. 5 days (95% CI 3, 9), *p* = 0.042]; they also experienced more deaths over the 28-day period (65 vs. 29%, *p* = 0.011). The multivariate regression analysis indicated that independent variables associated with SAD were THRR index [odds ratio (OR) = 5.770, 95% CI: 1.222–27.255; *p* = 0.027] and the mean value for rSO_2_ was < 55% (OR = 3.864, 95% CI: 1.026–14.550; *p* = 0.046).

**Conclusion:** Independent risk factors for SAD were mean cerebral oxygen saturation below 55% and cerebrovascular dysregulation (THRR < 1.09).

## Introduction

The sepsis-associated delirium (SAD) is regarded as a diffuse cerebral dysfunction, caused by inflammatory responses of the body to infections taking place with no discernible cause of central nervous system, i.e., infection. SAD may have a rapid onset and levels may fluctuate over time (1, 2). Research has demonstrated that pathogenesis of SAD can involve neuronal degeneration, neurotransmitter imbalance, abnormal cerebral perfusion, and neuroinflammation (3–6). Some researchers have proposed that there is a higher likelihood of SAD in patients experiencing cerebral edema, blood-brain barrier disruption, lower brain oxygen uptake, and variations in cerebral blood flow as a result of hemodynamic instabilities (7, 8). For sepsis patients, variations in cerebral autoregulation and perfusion can be visually detected by using transcranial Doppler (TCD) ultrasound. Significant links between SAD and cerebral vascular autoregulation disorders were revealed by Pfister et al. (9). Some study has found a close correlation between clinical manifestations of SAD and variations in cerebral hemodynamics found by using TCD ultrasound. Pierrakos et al. (10) found that pulsatility index (PI) on the first day could predict the positive Confusion Assessment Method for the Intensive Care Unit (CAM-ICU) test in sepsis patients (*p* < 0.01) and only on the first day, the mean blood velocity in the middle cerebral artery (MCA) and cerebral blood flow index (CBFi) was found to be lower in those patients with a high initial PI.

Hypoxemia and hypotension lead to an increase in neuronal apoptosis and, further, have direct associations with poor outcomes (11). Current methods for monitoring mixed venous oxygen saturation (SvO_2_) and central venous oxygen saturation (ScvO_2_) are non-continuous, demand invasive procedures, and do not permit pinpointed local brain tissue monitoring, meaning that rapid and effective identification of sepsis patients with higher risks of suffering brain/brain-tissue hypoxemia is not possible (12). Monitoring tissue oxygen saturation (StO_2_) and regional cerebral oxygen saturation (rSO_2_) by using near-infrared spectroscopy (NIRS) offers a noninvasive means of assessing cerebral and local tissue oxygen metabolism, offering continuous real-time data regarding the balance between demand and supply for oxygen. Additionally, rSO_2_ is a sensitive marker for global cerebral hypoperfusion (13). This study has demonstrated that monitoring of StO_2_ is a useful means of clinically evaluating sepsis/septic shock (14, 15).

In light of the above, this research employs TCD and StO_2_ monitoring for observation of the variations in the cerebral hemodynamic indices of the MCA and also variations in cerebral/peripheral StO_2_ for identification of elements that increase the risk of patients developing SAD.

## Materials and Methods

### Patients

This study selected patients suffering from septic shock accepted in the Department of Intensive Care Unit, Xiangya Hospital, Central South University between May 2018 and March 2019. This study represents a pilot observational cohort study; criteria for inclusion were set in alignment with the standard definition for sepsis 3.0. Every patient received treatment in accordance with the 2016 International Guidelines for the Treatment of Sepsis and Septic Shock (16), these being a mean arterial pressure (MAP) that reaches 65 mm Hg, with lactic acid normalization being the aim of the initial resuscitation procedures. All the patients in this study met the septic shock diagnostic criteria (17). Patients were excluded for any of the following criteria: aged below 18 years; past history of craniotomy or mental disorder; the presence of neurological disease (obvious intracranial lesions, e.g., intracranial infection, stroke, craniocerebral trauma, subarachnoid hemorrhage, cerebral hemorrhage); liver failure with suspicion of hepatic encephalopathy; pregnancy; abnormal outcomes of cervical vascular assessment with TCD or MRI/CT (e.g., carotid plaques or thrombosis with hemodynamically significant stenosis); and blood flow signals that could not be detected by using TCD within the time frame. Primary outcomes were either being discharged from ICU or developing delirium within 7 days of ICU admission; the secondary outcome was the mortality rate after 28 days.

### Ethical Approval/Informed Written Consent

This study was undertaken in accordance with accepted medical ethical standards. Approval was granted by the Xiangya Hospital, Central South University Ethics Committee (ethics no.: 2018101082); immediate relatives of patients provided informed written consent.

### Method for Evaluating Delirium

Delirium was diagnosed based on the CAM-ICU undertaken two times daily (10–11 a.m. and 4–5 p.m.) by qualified researchers in the ICU from the initial day of admission up to 7 days stay. Patients were allocated to the SAD cohort if they had two positive CAM-ICU screens carried out by the two investigators. Every septic shock patient was allocated the SAD or non-SAD group based on their suffering or not suffering delirium.

### Data Collection

General data were harvested for all the selected septic shock patients and also specific data regarding 28-day mortality rate (%), time spent in ICU (days), time spent on mechanical ventilation (days), continuous renal replacement therapy (CRRT), sedation/analgesia medication administered, the sequential (sepsis-related) organ failure assessment (SOFA) score, and the Acute Physiology and Chronic Health Evaluation II (APACHE II) score.

### Indicators of Circulating Hemodynamic Management

Indicators included urine output, total resuscitation fluid, norepinephrine dose level, central venous pressure (CVP), lactate clearance, and arterial/central venous blood gas indicators. The septic shock patient cohort for this research was given critical echocardiography within 60 min of being admitted measuring cardiac output (CO), left ventricular ejection fraction (LVEF), inferior vena cava diameter (IVCD), and inferior vena cava-collapse index (IVC-CI).

### Organ Function/Biochemical Markers

Monitoring of blood samples began as soon as the patient was admitted to ICU, which included organ function assessment with routine blood indicators, regulation indicators, and kidney and liver function indicators. The biomarker indicators associated with sepsis measured were central nervous system-specific protein (S100β), neuron-specific enolase (NSE), and procalcitonin (PCT).

### Transcranial Doppler Monitoring Index

Bilateral MCAs flow signals were obtained by using TCD ultrasound [Shenzhen Delikai (Nanshan District, Shenzhen, Guangdong Province) EMS-9A dual channel, 1.6 MHz TCD probe] once 6 h of initial resuscitation had been undertaken with the septic shock patient. The dynamic assessment for cerebral vascular autoregulation was completed, CBFi was calculated (CBFi = 10 × MAP/1.47 PI), and a record was made of PI (PI = Vs_MCA_−Vd_MCA_/Vm_MCA_), systolic velocity of MCAs (Vs_MCA_), mean blood flow velocity [mean velocity of MCAs (Vm_MCA_)], and the diastolic velocity of MCAs (Vd_MCA_). The THRR method was employed for assessing dynamic cerebral vascular autoregulation, i.e., MCA blood flow underwent stable lowering between 30 and 50% of baseline values employing confirmed carotid artery compression between 3 and 9 s; the blood flow velocity: baseline blood flow velocity ratio was also then measured. The THRR index above 1.09 is regarded as indicating dynamic cerebral vascular autoregulation function; if the levels fall below 1.09, this is regarded as indicating impairment to cerebral vascular autoregulation (18).

Tissue oxygen saturation levels were assessed by employing a noninvasive StO_2_ monitor (CAS Medical Systems, Inc.) for continuous monitoring of forehead rSO_2_ (large probe, 2.5 cm below skull detection depth) and thenar eminence StO_2_ (small probe, detection depth between 0.5 and 2 cm). The aforementioned values underwent continuous recording from several 60 mins once the septic shock patients had 6 h of initial resuscitation; uniform processing of the data to place in the later period. To find a level of rSO_2_ and StO_2_ data suited to comparison purposes, they were subjected to multiple threshold analysis (<50, <55, <60, and <65%) (19).

### Data Analysis

The data were analyzed by using the Statistical Package for the Social Sciences (SPSS) version 24.0 (SPSS Incorporation, Armonk, New York, USA). Normality testing of the data was undertaken. Continuous variable data that matched or were close to normal distribution were noted as mean ± SD (χ ± s). An independent Student's *t*-test was employed for comparing the two samples. Nonconforming data in terms of the normal distribution were noted as median [interquartile range (IQR)] and the two samples were compared employing the Wilcoxon rank-sum test. The chi-squared (*X*^2^) test was employed for comparisons of categorical data. A continuous correction methodology was used when the theoretical frequency was below five; an exact probability methodology was used when the theoretical frequency was below one. Independent predictors for delirium were detected by using the multivariate logistic regression analysis with predictor variables being chosen from the risk elements appearing in tables as *p* < 0.05. The correlation between the normal distribution variables mentioned above was analyzed by using the Pearson analysis method. *p* < 0.05 was regarded as having statistical significance.

## Results

**Figure 1:**
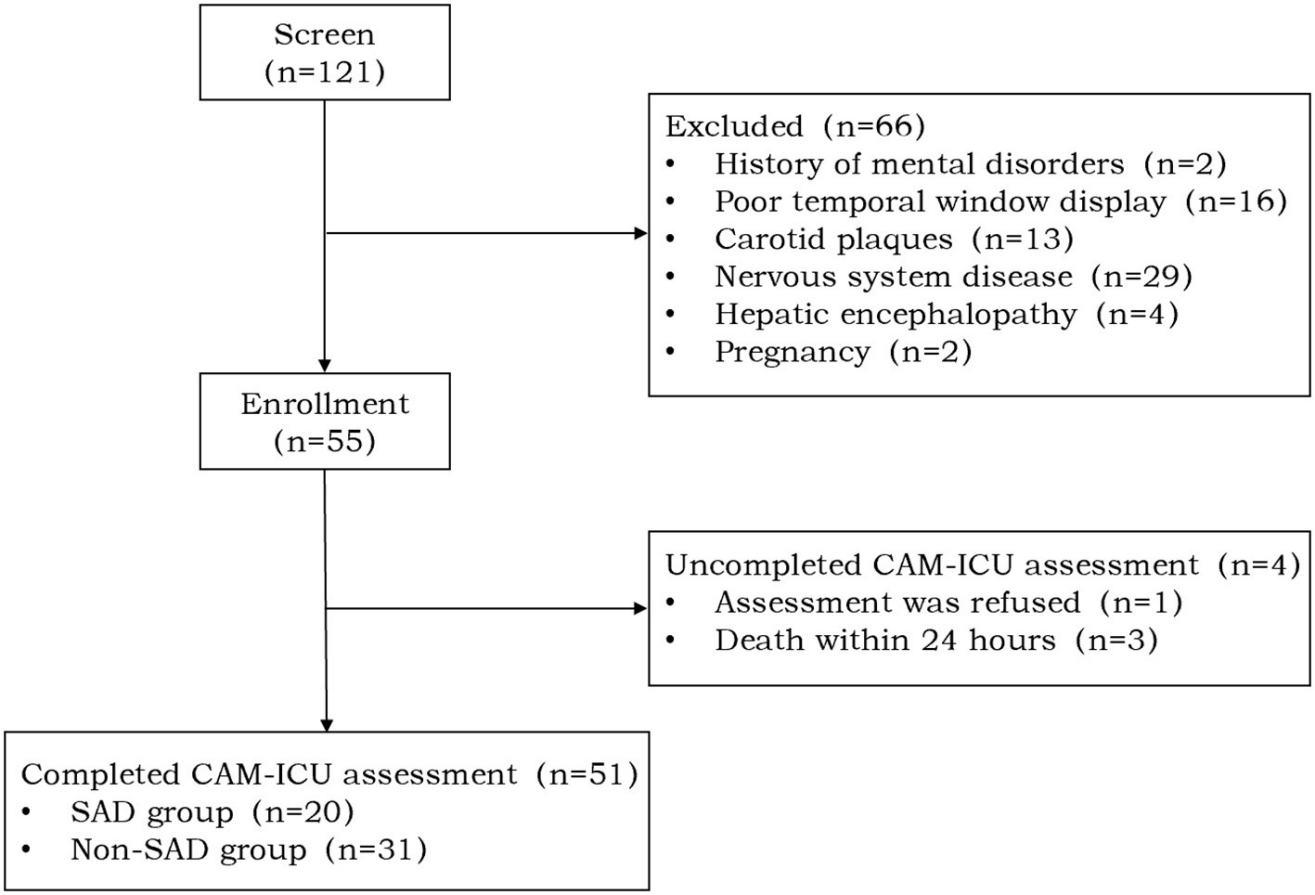
Study flow diagram. A total of 121 patients with septic shock were screened of which 66 patients met various exclusion criteria and four patients did not complete the Confusion Assessment Method for the Intensive Care Unit (CAM-ICU) assessment. A total of 51 patients were enrolled in our study of which 20 (39%) patients were positive for the CAM-ICU [sepsis-associated delirium (SAD) group] and 31 (61%) patients were negative for the CAM-ICU (non-SAD group).

A total of 121 septic shock patients were evaluated for this study; a total of 66 of them fulfilled one or more of the exclusion criteria and four patients did not undergo a full delirium evaluation. Finally having successfully completed the CAM-ICU evaluation, a cohort of 51 patients was selected for the research. About 39% experienced SAD (Figure 1).

### General Clinical Data/Diagnosis

Of the 51 patients suffering septic shock, 31 (61%) patients were men; the average age for the whole group was 53 ± 11 years. The APACHE II scores (21 ± 7 vs. 15 ± 6, *p* = 0.003) and the SOFA scores (12 ± 5 vs. 6 ± 3, *p* = 0.001) were higher for the individuals in the SAD group (Table 1). The median time for the overall length of mechanical ventilation was 5 (1, 5) days; the median time for stay in ICU was 6 (3, 12) days. The SAD group required more time on mechanical ventilation compared to the non-SAD group [5 (2, 6) vs. 1 (1, 4) days, *p* = 0.015] and a longer stay in ICU [9 (5, 20) vs. 5 (3, 9) days, *p* = 0.042]. For the group as a whole, 28-day mortality was 43%; mortality was significantly greater among those in the SAD group (65 vs. 29%, *p* = 0.011) (Table 1).

**Table 1:** Patient characteristics and comorbidities.

**Characteristic**	**The overall population** **(***n*** = 51)**	**SAD group** **(***n*** = 20)**	**Non-SAD group** **(***n*** = 31)**	***p*** **value**
Age (years)^a^	53 ± 11	56 ± 11	52 ± 14	0.239
Gender (man)^b^	31 (61)	13 (65)	18 (58)	0.620
BMI (kg/m^2^)^a^	22.59 ± 2.50	23.05 ± 2.48	22.29 ± 2.51	0.294
Education (years)^b^				0.884
<6	19 (37)	8 (40)	11 (36)	
6–9	13 (25)	4 (20)	9 (29)	
9–12	11 (22)	5 (25)	6 (19)	
12–16	8 (16)	3 (5)	5 (16)	
Hypertension^b^	14 (27)	6 (30)	8 (26)	0.743
Coronary heart disease^b^	8 (16)	4 (20)	4 (13)	0.775
Diabetes mellitus^b^	9 (18)	3 (15)	6 (19)	0.982
Temperature (°C)^a^	37.2 ± 0.9	37.2 ± 0.7	37.2 ± 1.0	0.936
Heart rate (beats/min)^a^	109 ± 17	112 ± 18	106 ± 16	0.276
Breaths (/min)^a^	22 ± 6	22 ± 6	22 ± 6	0.864
Pulse oxygen saturation (%)^a^	98 ± 3	97 ± 4	98 ± 3	0.211
Blood glucose (mmol/L)^a^	9.0 ± 4.5	9.4 ± 5.3	8.7 ± 3.9	0.597
MAP (mmHg)^a^	81 ± 14	82 ± 18	80 ± 11	0.687
SOFA^a^	8 ± 5	12 ± 5	6 ± 3	<0.001
APACHE II^a^	18 ± 7	21 ± 7	15 ± 6	0.003
Sedative^b^	25 (49)	11 (55)	14 (45)	0.493
Analgesic^b^	34 (67)	15 (75)	19 (61)	0.311
Detection rate of pathogenic bacteria^b^	29 (57)	13 (65)	16 (52)	0.242
CRRT^b^	16 (31)	9 (45)	7 (23)	0.092
Mechanical ventilation time (days)^c^	2 (1, 5)	5 (2, 6)	1 (1, 4)	0.002
ICU stay (days)^c^	6 (3, 12)	9 (5, 20)	5 (3, 9)	0.030
28-day mortality^b^	22 (43)	13 (65)	9(29)	0.011

a *Value shown as mean ± SD*.

b *Values shown as the number of patients (%)*.

c *Values shown as median [interquartile range (IQR)]*.

### Circulating Hemodynamics Indicators

The SAD group had a significantly lower oxygenation index (t6h) (230 ± 116 vs. 322 ± 121, *p* = 0.01), lower lactate clearance rate (t6h) (−0.36 ± 0.73 vs. −0.002 ± 0.35 mmol/L, *p* = 0.049), and higher blood lactate (Lac) (t6h) (5.2 ± 4.3 vs. 2.1 ± 1.3 mmol/L, *p* = 0.005). The critical echocardiography indicators showed no significant variations (*p* > 0.05). Additional File 1 (found in Supplementary Materials) gives greater detail.

### Organ Function/Biochemical Markers

The SAD group exhibited a significantly raised white blood cell count compared to the non-SAD group (21.38 ± 14.16 vs. 12.55 ± 11.61, *p* = 0.019); they also had significantly higher levels of NSE [18.24 (13.29, 27.08) vs. 9.55 (6.13, 18.11), *p* = 0.031] and blood urea nitrogen (BUN) [9.6 (5.7, 16.9) vs. 5.8 (2.8, 11.2) mmol/L, *p* = 0.046]. Additional File 2 (found in Supplementary Materials) gives greater detail.

### Transcranial Doppler-Detected Cerebral Hemodynamics in the MCA

The SAD group exhibited lower levels of V_dMCA_ compared to the non-SAD group (49.7 ± 20.3 vs. 61.9 ± 17.3 cm/s, *p* = 0.026) with a higher PI (0.98 ± 0.19 vs. 0.84 ± 0.20, *p* = 0.019). Across both the groups, the dynamic cerebral vascular dysfunction (THRR index < 1.09) was 22%; the SAD group was at a significantly higher level (40 vs. 10%, *p* = 0.01) (Table 2).

**Table 2:** Comparison of cerebral blood flow parameters of transcranial Doppler (TCD) between the sepsis-associated delirium (SAD) group and the non-SAD group.

**Variable**	**The overall population** **(***n*** = 51)**	**SAD group** **(***n*** = 20)**	**Non-SAD group** **(***n*** = 31)**	***p*** **value**
MAP (mmHg)^a^	81 ± 14	82 ± 18	80 ± 11	0.687
Vs_MCA_(cm/s)^a^	127.9 ± 37.9	122.0 ± 50.2	131.7 ± 27.7	0.436
Vm_MCA_(cm/s)^a^	79.2 ± 26.6	74.0 ± 29.7	82.7 ± 24.3	0.258
Vd_MCA_(cm/s)^a^	57.1 ± 19.3	49.7 ± 20.3	61.9 ± 17.3	0.026
PI^a^	0.89 ± 0.21	0.98 ± 0.19	0.84 ± 0.20	0.019
CBFi ^a^	648.73 ± 180.02	596.72 ± 196.38	682.28 ± 163.15	0.098
THRR index < 1.09^b^	11 (22)	8 (40)	3 (10)	0.010

a *Value shown as mean ± SD*.

b *Values shown as the number of patients (%)*.

### StO_2_ Monitoring

The SAD group had a lower mean rSO_2_ value compared to the non-SAD group (55 ± 7 vs. 60 ± 6, *p* = 0.01). Of the 51 patients, 16 patients had average rSO_2_ < 55% with significantly higher levels in the SAD group (50 vs. 19%, *p* = 0.021). Additionally, 15 patients had mean StO_2_ < 60%; again, a significantly greater number of the SAD group fall into this range (45 vs. 19%, *p* = 0.049) (Table 3).

**Table 3:** Tissue oxygen saturation (StO_2_).

	**The overall population** **(***n*** = 51)**	**SAD group** **(***n*** = 20)**	**Non-SAD group** **(***n*** = 31)**	***p***
rSO_2_ value (%)^a^
rSO_2_min	55 ± 7	53 ± 7	56 ± 6	0.055
rSO_2_max	62 ± 6	60 ± 6	63 ± 6	0.056
rSO_2_mean	58 ± 7	55 ± 7	60 ± 6	0.010
Number of patients^b^
rSO_2_mean < 60%	29 (57)	14 (70)	15 (48)	0.128
rSO_2_mean < 55%	16 (31)	10 (50)	6 (19)	0.021
rSO_2_mean < 50%	7 (14)	5 (25)	2 (6)	0.144
StO_2_value (%)^a^
StO_2_min	63 ± 8	62 ± 9	64 ± 7	0.391
StO_2_max	70 ± 7	69 ± 9	71 ± 6	0.373
StO_2_mean	67 ± 7	65 ± 9	68 ± 6	0.335
Number of patients^b^
StO_2_mean < 65%	16 (31)	9 (45)	7 (23)	0.092
StO_2_mean < 60%	15 (29)	9 (45)	6 (19)	0.049
StO_2_mean < 55%	6 (12)	4 (20)	2 (6)	0.307

a *Value shown as mean ± SD*.

b *Values shown as the number of patients (%)*.

*The SAD group vs. the non-SAD group*.

### Multivariate Analysis of SAD Risk Factors

In the multivariate regression analysis for risk factors focused on SAD brain parameters, the logistic regression analysis demonstrated that several independent risks were SAD predictors: these included mean rSO_2_ < 55% [odds ratio (OR) = 3.864, 95% CI: 1.026 to 14.550, *p* = 0.046] and the THRR index < 1.09 (OR = 5.77, 95% CI: 1.222–27.255, *p* = 0.027) (Table 4).

**Table 4:** The multivariate regression analysis of risk factors focuses on the brain parameters for SAD.

**Variable**	**B**	**S.E**.	**Wals**	***p***	**Exp(B)**	**95% C.I. for EXP (B)**	
						**upper**	**lower**
PI	1.160	2.109	0.303	0.582	3.191	0.051	199.021
V_dMCA_	−0.035	0.024	2.177	0.140	0.965	0.921	1.012
THRR index < 1.09	1.753	0.792	4.896	0.027	5.770	1.222	27.255
rSO2 mean	−0.083	0.058	2.045	0.153	0.921	0.822	1.031
rSO2mean < 55%	1.352	0.676	3.993	0.046	3.864	1.026	14.550

*Sig., significance; EXP(B), estimated odds ratio (OR)*.

## Discussion

Imbalances in cerebral oxygen metabolism and impairment of cerebral hemodynamics are key factors in whether or not patients develop SAD. This study confirms that there are high rates of SAD in septic shock patients and it has associations with poor outcomes. It has been shown that independent risk factors for the condition are mean cerebral oxygen saturation below 55% and cerebral vascular autoregulation dysfunction (THRR index < 1.09).

The first point to note in this study is that 39% of septic shock patients were suffering from SAD (20/51); this group had the higher APACHE II and the SOFA scores, needed more time on mechanical ventilators and longer stays in ICU, and their 28-day survival rate was significantly lower. This demonstrated that SAD has a close correlation with severe illness and poor outcomes. So, it is important to identify patients who were at risk for developing delirium. Mori et al. (20) showed a high incidence of ICU delirium associated with older age, use of sedatives and analgesics, emphasizing the need for relevant nursing care to prevent and identify early patients presenting these characteristics. Tse et al. (21) have suggested that delirium following cardiac surgery is influenced by age and several underlying diseases/conditions. Drugs and drug regimens for the prevention of delirium have not currently existed, so clinicians should concentrate on the non-medication interventions; these include keeping lines of communication open, identifying any psychological difficulties patients have early, keeping patients in touch with their family promoting healthy sleep, keeping noise to a minimum, and attempting to get patients active early (22).

Second, it was demonstrated that with septic shock patients, cerebral vascular autoregulation dysfunction is an independent risk factor. This would suggest that 6 h following initial resuscitation, patients experiencing SAD have a fall in cerebral vascular compliance and cerebral perfusion. These fluctuations could have an additional impact on cerebral vascular autoregulation. Cerebral oxygen metabolism and cerebral hemodynamic disorders have a key part in developing SAD, which should inform clinical practice in managing hemodynamics with a focus on cerebral perfusion. There is a close correlation between the central nervous system dysfunction seen in sepsis and cerebral hypoperfusion as a result of a fall in cerebral blood flow. Cerebral perfusion can be evaluated through monitoring of systolic, diastolic, and mean blood flow velocity in the anterior, middle, and posterior cerebral arteries by using TCD ultrasound (23). TCD also offers an indirect means of assessing cerebral circulation, which includes cerebral vascular autoregulation. With impairment in cerebral vascular autoregulation, fluctuations in perfusion pressure can cause cerebral congestion or ischemia resulting in nerve damage, which can have a negative impact on outcomes. In 1997, Smielewski et al. (24) discovered a correlation between a vanished transient cerebral congestion response rate (THRR) and poor outcomes for patients who had suffered serious craniocerebral injuries. While the value of brain imaging cannot be completely substituted for TCD and THRR index evaluation, they provide a means of continually monitoring cerebrovascular autoregulation in the early stages of treatment for sepsis/septic shock patients (25). Early identification of cerebrovascular autoregulation dysfunction will allow clinical interventions to take place earlier.

Third, the oxygen saturation of the brain has to be dynamically monitored in real time if cerebral perfusion is to be optimized by determining any oxygen supply/demand incompatibilities. This study demonstrated that the group with SAD had lower mean rSO_2_ values than those who did not; the multivariate regression illustrated that for septic shock patients, a mean rSO_2_ < 55% increases the likelihood of delirium. It appears that generally, patients with SAD have low levels of cerebral oxygen saturation. Discrepancies between oxygen consumption and cerebral oxygen supply are key to patients developing SAD and so septic shock patients must be continually monitored for cerebral oxygen saturation levels. As with heart rate, respiration, blood pressure, and pulse oxygen saturation, cerebral oxygen saturation level should become a standard monitoring indicator. The study has shown that intraoperative regional rSO_2_ levels below 40% represent a significant independent risk factor for cognitive impairment following cardiac surgery (26). According to De Tournay-Jette et al. (27), it has been reported that subjects with a rSO_2_ below 50% in the course of surgery had greater levels of cognitive dysfunction postoperatively 4 to 7 days after surgery (*p* = 0.04) and that patients who have experienced a reduction in relation to their baseline rSO_2_ of more than 30% were more likely to display cognitive impairment 1 month after their surgery (*p* = 0.03). When oxygen saturation is monitored, it offers essential data regarding brain tissue levels of oxygen that can be impacted by several diseases (28).

Finally, mean StO_2_ values varied from 60 to 74% following the initial resuscitation of septic shock patients. While there are normalized global parameters for hemodynamics, optimization of them may result in changes in regional perfusion and microcirculation. If these changes persist, a poor outcome is more likely. StO_2_ may function as an early alert to the fact that tissue hypoxia/low perfusion is beginning (29).

This study has some limitations. First, the ability of the technician can influence how effective TCD ultrasound can be. Second, evaluation was only undertaken of variations in cerebral hemodynamics and oxygen saturation in cerebral and peripheral tissue 6 h after initial resuscitation; we did not combine this with neurophysiological findings or neuroimaging. Third, patients with unattainable TCD flow spectrum or with a carotid plaque were excluded, which will affect its application to the entire septic shock patient population. As this study was observational in design and only involved a small patient cohort, randomized controlled trials (RCTs) on a larger scale may be necessary to verify our findings.

## Conclusion

Patients with SAD have a close correlation with poor outcomes. Independent risk factors for SAD were mean cerebral oxygen saturation below 55% and cerebrovascular dysregulation (THRR < 1.09). This would justify the provision of RCT with adequate power for patient treatment in the future.

## Data Availability Statement

The raw data supporting the conclusions of this article will be made available by the authors, without undue reservation.

## Ethics Statement

This prospective observation study was undertaken at an academic hospital in an urban area of China. All patients involved gave written informed consent for their participation in this study. The Ethics Committee of Xiangya Hospital of Central South University approved all elements of this research (Ethics no: 2018101082). The patients/participants provided their written informed consent to participate in this study.

## Author Contributions

QF undertook the statistical analysis, researched the scientific literature, and drafted the manuscript. MA, LH, and QP assisted in data collection. YA made contributions to the initial concept, design, and data interpretation. LZ also made contributions to the initial concept, design, data interpretation, editing, revision of the manuscript, and supervised this study. The manuscript has been reviewed and approved by all the authors.

## Funding

This study benefitted from support in the form of grants from the Chinese National Natural Science Foundation, China (81873956).

## Conflict of Interest

The authors declare that the research was conducted in the absence of any commercial or financial relationships that could be construed as a potential conflict of interest.

## Publisher's Note

All claims expressed in this article are solely those of the authors and do not necessarily represent those of their affiliated organizations, or those of the publisher, the editors and the reviewers. Any product that may be evaluated in this article, or claim that may be made by its manufacturer, is not guaranteed or endorsed by the publisher.
