# Supplementary material for: Relationship Between Cerebral Hemodynamics, Tissue Oxygen Saturation, and Delirium in Patients With Septic Shock: A Pilot Observational Cohort Study
Source: Front Med (Lausanne). 2021 Nov 26;8:641104. doi: 10.3389/fmed.2021.641104 (PMC8660998; doi:10.3389/fmed.2021.641104)
Supplement: Supplementary file 1 [file Data_Sheet_1.docx]

Additional file 1. Comparison of organ function and biomarkers between sepsis-associated delirium (SAD) group and non-SAD group.

| Variable | The overall population  （n=51） | SAD group  （n=20） | Non-SAD group (n=31) | *P* value |
| --- | --- | --- | --- | --- |
| WBC（x10^9^/L）^a^ | 16.01±13.26 | 21.38±14.16 | 12.55±11.61 | 0.019 |
| Hb (g/L) ^a^ | 93.10±23.66 | 93.45±28.43 | 92.87±20.52 | 0.933 |
| Hct（%）^a^ | 28.62±7.20 | 28.72±8.80 | 28.56±6.11 | 0.939 |
| PLT (/L) ^a^ | 145.78±110.97 | 126.70±12.02 | 158.10±116.34 | 0.329 |
| ALB (g/L) ^a^ | 26.04±5.59 | 25.76±5.89 | 26.22±5.47 | 0.780 |
| TBIL (umol/L,)^b^ | 15.0[7.2,29.2] | 20.9[9.6,84.3] | 13.9[7.0,27.3] | 0.222 |
| DBIL (umol/L,) ^b^ | 9.0[2.9,18.2] | 10.8[4.2,38.0] | 18.2[7.4,51.7] | 0.246 |
| ALT (U/L,) ^b^ | 21.6[11.9,56.5] | 43.3[14.8,69.1] | 19.8[10.7,31.5] | 0.260 |
| AST (U/L,) ^b^ | 28.7[21.0,81.0] | 52.5[21.0,108.3] | 26.1[20.2,54.0] | 0.333 |
| BUN (mmol/L,) ^b^ | 7.0[4.6,12.6] | 9.6[5.7,16.9] | 5.8[2.8,11.2] | 0.046 |
| UA (umol/L,) ^b^ | 295.0[200.8,410.7] | 293.5[197.7,258.5] | 324.0[229.7,547.4] | 0.406 |
| Cr (mmol/L) ^a^ | 146.61±92.69 | 157.83±100.79 | 139.37±88.02 | 0.493 |
| APTT ^a^ | 41.09±12.15 | 42.29±11.05 | 40.32±12.93 | 0.565 |
| INR ^a^ | 1.40±0.55 | 1.55±0.81 | 1.30±0.25 | 0.199 |
| PCT(µg/L]) ^b^ | 17.56[4.75,84.03] | 17.21[6.83,79.76] | 17.97[4.09,83.85] | 0.869 |
| NSE (µg/L]) ^b^ | 13.72[8.18,21.26] | 18.24[13.29,27.08] | 9.55[6.13,18.11] | 0.031 |
| S100β(µg/L,) ^b^ | 0.27[0.13,0.54] | 0.34[0.23,0.54] | 0.24[0.10,0.54] | 0.780 |

a value shown as mean ± standard deviation; b values shown as median [IQR].

WBC,white blood cell; Hb,hemoglobin; Hct,hematocrit; PLT, platelet; ALB,albumin; TBIL,total bilirubin; DBIL,direct bilirubin; ALT,[glutamic-pyruvic](javascript:;) [transaminase](javascript:;); AST,[glutamic](javascript:;) [oxaloacetic](javascript:;) [transaminase](javascript:;); BUN,[urea](javascript:;) [nitrogen](javascript:;); UA,[uric](javascript:;) a[cid](javascript:;); Cr,creatinine; APTT,activated partial thromboplastin time; INR,international normalized ratio; PCT,procalcitonin; NSE,neuron specific enolase; S100β,central nervous system specific protein.

Additional file 2. Circulation hemodynamic indexes at study enrollment (t0h) and 6 hours post-resuscitation (t6h).

| Variable | The overall population  （n=51） | SAD group  （n=20） | Non-SAD group(n=31) | *P* value |
| --- | --- | --- | --- | --- |
| pH ^a^ |  |  |  |  |
| t0h | 7.37±1.00 | 7.37±1.00 | 7.37±0.90 | 0.986 |
| t6h | 7.37±0.90 | 7.36±0.97 | 7.37±0.83 | 0.663 |
| Lac (mmol/L) ^a^ |  |  |  |  |
| t0h | 3.2±3.2 | 4.5±4.5 | 2.4±1.6 | 0.056 |
| t6h | 3.3±3.2 | 5.2±4.3 | 2.1±1.3 | 0.005 |
| PO_2_ (mmHg) ^a^ |  |  |  |  |
| t0h | 110±54 | 109±55 | 111±54 | 0.921 |
| t6h | 120±42 | 106±38 | 129±42 | 0.051 |
| PCO_2_ (mmHg) ^a^ |  |  |  |  |
| t0h | 39±7 | 45±7 | 34±8 | 0.327 |
| t6h | 36±7 | 34±7 | 37±6 | 0.079 |
| FiO_2_/PO_2_^a^ |  |  |  |  |
| t0h | 272±129 | 271±130 | 272±132 | 0.980 |
| t6h | 286±127 | 230±116 | 322±121 | 0.010 |
| Pcv-aCO_2_（mmHg）^a^ |  |  |  |  |
| t0h | 8.1±3.8 | 9.8±4.7 | 7.3±3.2 | 0.184 |
| t6h | 6.5±3.4 | 7.3±4.4 | 6.0±2.6 | 0.349 |
| ScvO_2_（%）^a^ |  |  |  |  |
| t0h | 65±9 | 64±3 | 65±11 | 0.696 |
| t6h | 68±12 | 65±14 | 69±11 | 0.317 |
| CVP（mmHg）^a^ |  |  |  |  |
| t0h | 6.4±3.7 | 5.6±3.4 | 6.8±3.9 | 0.473 |
| t6h | 6.4±3.9 | 5.9±4.7 | 6.7±3.4 | 0.569 |
| Lactate clearance rate  (mmol/L) ^a^ | -0.14±0.56 | -0.36±0.73 | -0.002±0.35 | 0.049 |
| Noradrenaline（ug/kg*min）^b^ | 0.40[0.20,0.82] | 0.67[0.35,1.00] | 0.33[0.20,0.70] | 0.137 |
| Total liquid input（ml，t6h，M[IQR]）^b^ | 1670[1250,2200] | 1570[1250,1950] | 2000[1250,2400] | 0.106 |
| Total colloid input（ml，t6h，M[IQR]）^b^ | 100[13,144] | 100[13,100] | 100[50,150] | 0.939 |
| Urine volume(ml，t6h，M[IQR]) ^b^ | 415[155,800] | 450[163,700] | 385[155,970] | 0.462 |
| CO (L/min, M[IQR]）^b^ | 5.03[4.20,6.21] | 5.10[4.38,6.35] | 4.90[4.20,6.20] | 0.289 |
| LVEF %^a^ | 58±16 | 54±21 | 61±10 | 0.248 |
| IVCD (mm) ^a^ | 16±7 | 17±8 | 15±5 | 0.314 |
| IVC-CI (%) ^a^ | 25.94±18.55 | 25.00±18.54 | 26.67±19.05 | 0.806 |

a value shown as mean ± standard deviation; b values shown as median [IQR].

t0h, immediately after admission to critical care; t6h,6h post initial resuscitation in ICU. Lac,lactic acid; Pcv-aCO2 ,central venous to arterial carbon dioxide partial pressure difference; ScVO2 ,central venous oxygen saturation; PaO2, partial pressure of oxygen; PaCO2,partial pressure of carbon dioxide; CVP,central venous pressure; CO,cardiac output; LVEF%,left ventricular ejection fraction; IVCD,inferior vena cava diameter; IVC-CI,inferior vena cava collapse index.
